# Biological Mechanisms Underlying Physical Fitness and Sports Performance: An Editorial

**DOI:** 10.3390/biology11101425

**Published:** 2022-09-29

**Authors:** Georgian Badicu, Filipe Manuel Clemente, Eugenia Murawska-Cialowicz

**Affiliations:** 1 Department of Physical Education and Special Motricity, Transilvania University of Brasov, 500068 Brasov, Romania; 2 Escola Superior Desporto e Lazer, Instituto Politécnico de Viana do Castelo, Rua Escola Industrial e Comercial de Nun’Álvares, 4900-347 Viana do Castelo, Portugal; 3 Research Center in Sports Performance, Recreation, Innovation and Technology (SPRINT), 4960-320 Melgaço, Portugal; 4 Instituto de Telecomunicacoes, Delegacao da Covilha, 1049-001 Lisboa, Portugal; 5 Physiology and Biochemistry Department, Wroclaw University of Health and Sport Sciences, 51-612 Wroclaw, Poland

In general, the concept of a mechanism in biology has three distinct meanings. It may refer to a philosophical thesis about the nature of life and biology, to the internal workings of a machine-like structure, or to the causal explanation of a particular phenomenon [1]. 

Understanding the biological mechanisms that justify acute and chronic physiological responses to exercise interventions determines the development of training principles and training methods. A strong understanding of the effects of exercise in humans may help researchers to identify what causes specific biological changes and to properly identify the most adequate processes for implementing a training stimulus [1].

Despite the significant body of knowledge regarding the physiological and physical effects of different training methods (based on load dimensions), some biological causes of those changes are still unknown. Additionally, few studies have focused on natural biological variability in humans and how specific human properties may underlie different responses to the same training intervention. Thus, more original research is needed to provide plausible biological mechanisms that may explain the physiological and physical effects of exercise and training in humans.

In this Special Issue, we discuss/demonstrate the biological mechanisms that underlie the beneficial effects of physical fitness and sports performance, as well as their importance and their role in/influences on physical health.

A total of 28 manuscripts are published here, of which 25 are original articles, two are reviews, and one is a systematic review.

Two papers are on neuromuscular training programs (NMTs), training monotony (TM), and training strain (TS) in soccer players [2,3]; five articles provide innovative findings about testosterone and cortisol [4,5], gastrointestinal hormones [6], spirulina [7], and concentrations of erythroferrone (ERFE) [8]; another five papers analyze fitness and its association with other variables [7,9,10,11,12]; three papers examine body composition in elite female soccer players [2], adolescents [6], and obese women [7]; five articles examines the effects of high-intensity interval training (HIIT) [7,10,13,14,15]; one paper examines the acute effects of different levels of hypoxia on maximal strength, muscular endurance, and cognitive function [16]; another article evaluates the efficiency of using vibrating exercise equipment (VEE) compared with using sham-VEE in women with CLBP (chronic low-back pain) [17]; one article compares the effects of different exercise modes on autonomic modulation in patients with T2D (type 2 diabetes mellitus) [14]; and another paper analyzes the changes in ABB (acid–base balance) in the capillaries of kickboxers [18]. Other studies evaluate: the effects of resistance training on oxidative stress and muscle damage in spinal cord-injured rats [19]; the effects of muscle training on core muscle performance in rhythmic gymnasts [20]; the physiological profiles of road cyclist in different age categories [21]; changes in body composition during the COVID-19 [22]; a mathematical model capable of predicting 2000 m rowing performance using a maximum-effort 100 m indoor rowing ergometer [23]; the effects of ibuprofen on performance and oxidative stress [24]; the associations of vitamin D levels with various motor performance tests [12]; the level of knowledge on FM (Fibromyalgia) [25]; and the ability of a specific BIVA (bioelectrical impedance vector analysis) to identify changes in fat mass after a 16-week lifestyle program in former athletes [26]. Finally, one review evaluates evidence from published systematic reviews and meta-analyses about the efficacy of exercise on depressive symptoms in cancer patients [27]; another review presents the current state of knowledge on satellite cell-dependent skeletal muscle regeneration [28]; and a systematic review evaluates the effects of exercise on depressive symptoms among women during the postpartum period [29].

## Data Availability

Not applicable.
